# Effects of complex spa therapy in patients with osteoarthritis of the spine receiving treatments in health resorts in south-eastern Poland

**DOI:** 10.1038/s41598-022-18046-6

**Published:** 2022-08-29

**Authors:** Jolanta Zwolińska, Monika Gąsior

**Affiliations:** 1 St Queen Jadwiga’s Regional Clinical Hospital No. 2 in Rzeszow, ul. Lwowska 60, 35-301 Rzeszów, Poland; 2 Institute of Health Sciences, Medical College, University of Rzeszow, al. Rejtana 16A, 35-959 Rzeszów, Poland; 3 Centre for Innovative Research in Medical and Natural Sciences, University of Rzeszow, Poland, ul. Warzywna 1a, 35-310 Rzeszów, Poland

**Keywords:** Health care, Rheumatology

## Abstract

Management of patients with degenerative diseases commonly comprises health-resort based treatment programs, including spa therapies, balneotherapy as well as terrain therapy making use of microclimate factors. The study was designed to assess short- and long-term effects of spa therapy administered to patients with osteoarthritis of the spine who received treatment in health resorts located in Poland. The study involved 102 patients receiving treatment in health resorts, a group of subjects receiving outpatient treatment (100 patients) and a group receiving no therapy (100 patients). The assessment survey included: Pain VAS and Laitinen, LISAT-9 and HAQ-20 questionnaires. The assessments were carried out three times: at the start of the therapy program, as well as one month and six months after the end of the program. Short-term effects showed statistically significant improvement in all the outcome measures in spa group and outpatient treatment group. The long-term effects showed statistically significant improvement in all the outcome measures in spa group only. In conclusion spa therapy reduces pain, improves functional efficiency and increases the level of life satisfaction in patients with osteoarthritis of the spine. Its effects are sustained for at least six months. Spa therapy is more effective long-term, than outpatient treatment.

Trial registration: The study was registered at Clinical Trials: NCT03974308. First registration: 04/06/2019.

## Introduction

Pain syndromes in the spinal region are among the most common musculoskeletal complaints and may lead to disability and decreased quality of life^1,2^. Spa therapy programs comprise complex medical care procedures provided in a health resort, and are widely applied in management of musculoskeletal disorders^3,4^. This is a good alternative for patients with osteoarthritis, allowing these individuals to return to work, hence spa therapy potentially leads to lower absences at work. It has also been shown to reduce patients’ need for pharmacotherapy, which generates high costs in addition to possible undesired side-effects^5–8^. Spa therapy may also be applied jointly with conventional treatment methods, such as pharmacotherapy, physiotherapy and surgical procedures^1^. The basic therapeutic factors applied in spa therapy programs include natural materials and microclimate specific to a given health resort, as well as adequately designed exercise, including terrain therapy and other additional free-time activities as well as psychotherapy^9,10^. A stay at a health resort is also associated with lifestyle changes related to dietary habits and physical activity^9^. Isolation from work, social activity and family life contributes to more effective relaxation^10,11^. In the case of older individuals, spa therapy administered away from their place of residence may, for psychological reasons, lead to better effects, compared to treatment applied at home^12^.

Balneotherapy is a basic element of health-resort based programs, and is applied to prevent and treat neurological, orthopaedic and rheumatic disorders and in the related rehabilitation programs^3,9,13–15^. Its beneficial effects are linked to physical and chemical factors, yet the mechanism of its action is not fully understood^13^. Balneotherapeutic methods, specific to each health resort, produce anti-inflammatory and angiogenic effects and stimulate tissue regeneration^16,17^. Balneotherapeutic procedures are frequently applied by doctors and they are favoured by patients with chronic low back pain^18,19^.

Some researchers emphasise a need for studies assessing effectiveness of spa therapy based on long-term observation^18,20^. If effectiveness of various balneotherapeutic factors is confirmed, it will be easier to adequately design treatment and rehabilitation programs, and it will be possible to increase financing of spa therapy^20^.

Novelty of our study lies in the fact that we enrolled relatively large groups of subjects, and we compared group of individuals receiving spa therapy in health resorts, group of subjects receiving outpatient treatment at the place of residence, and control group receiving no treatment. Furthermore, we applied a variety of research tools, and the final assessment of effects was carried out as a follow-up after six months. Purpose: The study was designed to assess short- and long-term effects of spa therapy administered to patients with osteoarthritis of the spine who received treatment in health resorts located in south-eastern Poland.

## Results

### Study group

**Figure 1:**
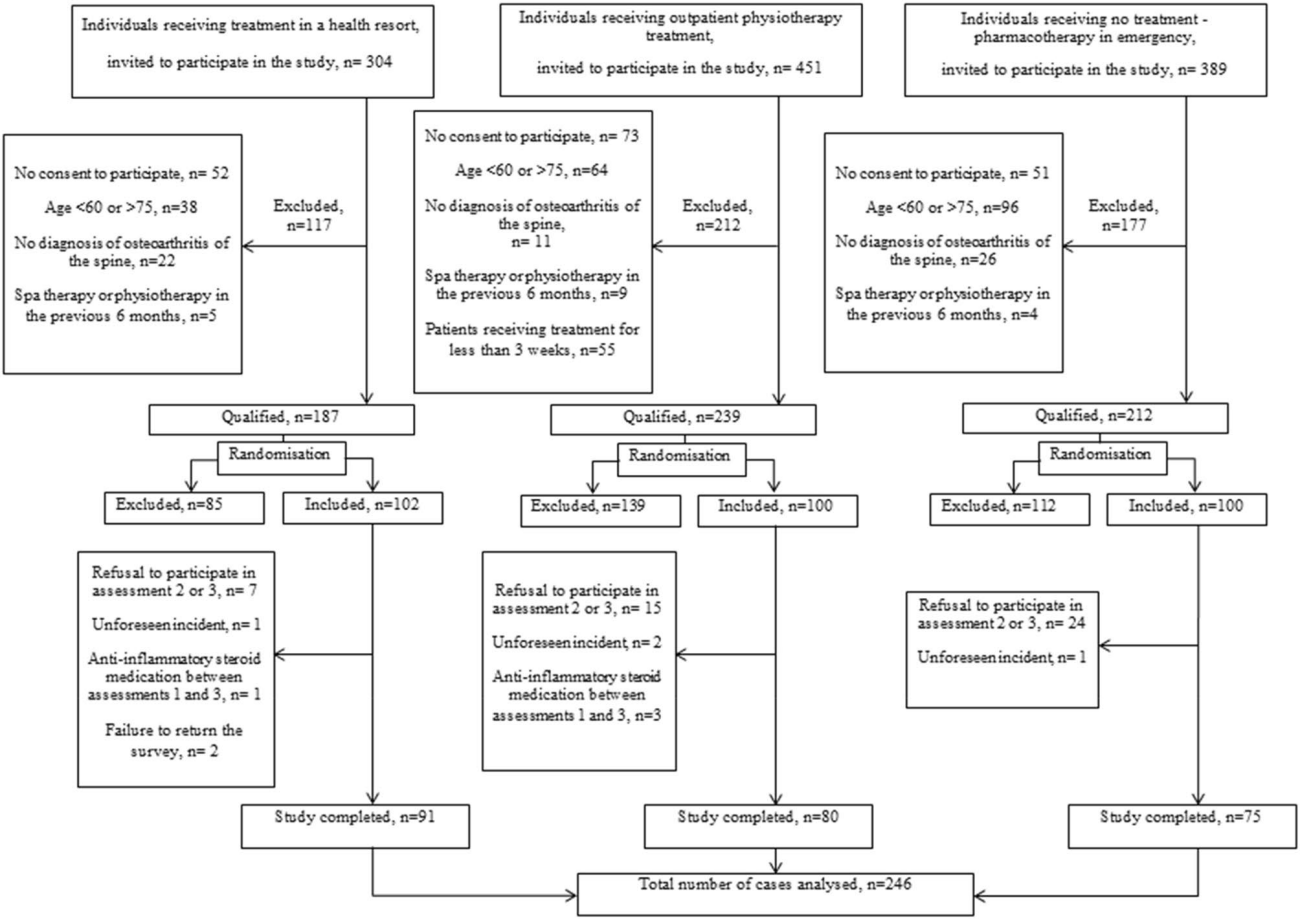
Flow diagram of study population.

Ultimately, 246 subjects participated in all three surveys; 56 individuals did not take part in the follow-up assessments for various reasons. The analyses took into account scores obtained in the survey by the spa group (91 subjects), outpatient treatment group (80 subjects) and control group (75 subjects) (Fig. 1).

Statistically significant differences were identified between the groups as regards the location of the complaints, place of residence and BMI value. Pain was most frequently reported in the lumbosacral spine (overall in all the groups-191 subjects). Majority of the subjects were residents of urban areas (overall-148 subjects), and abnormal body weight was identified in 68% of all the subjects. Those receiving spa therapy on average had experienced the related symptoms for a period four years longer, compared to outpatient treatment group (Table 1).

**Table 1:** Characteristics of the groups studied.

Characteristics	Spa group			Outpatient treatment group			Control group			*p*_1_	
	*N*	%		*N*	%		*N*	%			
**Sex**											
Male	39	42.9		31	38.8		27	36.0		0.6595	
Female	52	57.1		49	61.3		48	64.0			
**Type of occupation**											
Blue-collar worker	38	41.8		38	47.5		30	40.0		0.8918	
White-collar worker	49	53.8		38	47.5		41	54.7			
Mixed	4	4.4		4	5.0		4	5.3			
**Location of pain**											
L-S	82	90.1		62	77.5		47	62.7		0.0001***	
C	50	54.9		16	20.0		39	52.0		0.0000***	
Th	11	12.1		25	31.3		8	10.7		0.0007***	
**Place of residence**											
Rural area	26	28.6		47	58.8		25	33.3		0.0001***	
Urban area	65	71.4		33	41.3		50	66.7			
**Classification based on BMI**											
Normal	17	18.7		22	27.5		40	53.3		0.0000***	
Overweight	40	44.0		39	48.8		27	36.0			
Obesity	34	37.4		19	23.8		8	10.7			

	Mean	Median	SD	Mean	Median	SD	Mean	Median	SD	*p*_2_	
Age [years]	66.7	66	4.6	68.0	68	4.7	67.1	68	4.6	0.1839	
Duration of symptoms [years]	15.8	13	8.7	11.8	8	10.0	13.9	12	6.8	0.0004***	

*p*_1_—Probability value calculated using chi-square test of independence.

*p*_2_—Probability value calculated using Kruskal–Wallis test.

### Effects of spa therapy

Statistically significant differences were found between the three groups in all the measures. The controls, who were not receiving any systematic treatment, reported the least severe pain, and the highest efficiency and life satisfaction (Table 2).

**Table 2:** Mean psychometric measures in the specific examinations.

Quality of life measures	Spa group	Outpatient treatment group	Control group	*p*
	Mean (95% c.i.)			
**First assessment**				
Laitinen questionnaire	6.18 (5.64; 6.71)	6.60 (6.05; 7.15)	5.51 (5.10; 5.92)	0.0227*
Pain VAS	5.51 (5.08; 5.93)	6.19 (5.86; 6.52)	5.21 (4.79; 5.63)	0.0018**
LISAT-9	4.63 (4.47; 4.78)	4.36 (4.22; 4.50)	4.74 (4.58; 4.90)	0.0003***
HAQ-20	0.95 (0.83; 1.07)	1.13 (1.00; 1.25)	0.73 (0.59; 0.86)	0.0002***
**Second assessment**				
Laitinen questionnaire	3.68 (3.13; 4.23)	4.89 (4.29; 5.49)	5.29 (4.90; 5.69)	0.0000***
Pain VAS	3.85 (3.30; 4.39)	4.84 (4.43; 5.25)	4.87 (4.47; 5.26)	0.0038**
LISAT-9	4.71 (4.55; 4.87)	4.51 (4.38; 4.64)	4.74 (4.59; 4.90)	0.0206*
HAQ-20	0.72 (0.60; 0.84)	0.97 (0.85; 1.08)	0.73 (0.59; 0.86)	0.0031**
**Third assessment**				
Laitinen questionnaire	2.77 (2.30; 3.24)	5.73 (5.09; 6.36)	5.59 (5.15; 6.03)	0.0000***
Pain VAS	2.74 (2.29; 3.19)	5.91 (5.55; 6.27)	5.04 (4.62; 5.46)	0.0000***
LISAT-9	4.94 (4.80; 5.08)	4.38 (4.25; 4.52)	4.72 (4.57; 4.86)	0.0000***
HAQ-20	0.57 (0.47; 0.67)	1.10 (0.99; 1.20)	0.75 (0.62; 0.87)	0.0000***

*p*—Probability value calculated using Kruskal–Wallis test.

Results of post-hoc tests show that statistically significant differences were related mainly to outpatient treatment group and control group, and in the case of VAS and LISAT-9 scores also to spa group and outpatient treatment group. Group 2 differed in terms of the baseline results from the two other groups. The second and third examination showed statistically significant differences between spa group and outpatient treatment group in all the measures taken into account. Differences of very high statistical significance were identified between spa group and outpatient treatment group during the third examination (Table 3).

**Table 3:** Results of multiple comparison test in the specific examinations.

Quality of life measures	Post-hoc comparison results (*p*)		
	1 versus 2	1 versus 3	2 versus 3
**First assessment**			
Laitinen questionnaire	0.3879	0.5748	0.0200*
Pain VAS	0.0338*	0.9653	0.0022**
LISAT-9	0.0058**	1.0000	0.0005***
HAQ-20	0.2089	0.0384*	0.0001***
**Second assessment**			
Laitinen questionnaire	0.0052**	0.0000***	0.3390
Pain VAS	0.0123*	0.0168*	1.0000
LISAT-9	0.0386*	1.0000	0.0584
HAQ-20	0.0066**	1.0000	0.0150*
**Third assessment**			
Laitinen questionnaire	0.0000***	0.0000***	1.0000
Pain VAS	0.0000***	0.0000***	0.0490*
LISAT-9	0.0000***	0.0721	0.0019**
HAQ-20	0.0000***	0.0693	0.0002***

1—Spa group, 2—Outpatient treatment group, 3—Control group.

Assessment of the short-term effects showed positive changes in the measures in both the spa group and in the outpatient treatment group. In the control group a slight improvement was observed only in the scores on Laitinen scale and VAS. Results of post-hoc comparisons showed no statistically significant differences between spa group and outpatient treatment group in any measures taken into account. On the other hand, the tests showed significant differences between these two groups and the control group. As an exception no difference was identified between the outpatient treatment group and the controls in LISAT-9 scores (Table 4).

**Table 4:** Short-term effects in the groups studied.

Short-term therapeutic effect	Spa group (1)	Outpatient treatment group (2)	Control group (3)	*p*
	Mean therapeutic effect (95% c.i.)			
Laitinen questionnaire	− 2.49 (− 3.05; − 1.94)	− 1.71 (− 2.09; − 1.33)	− 0.21 (− 0.54; 0.11)	0.0000***
Pain VAS	− 1.66 (− 2.23; − 1.09)	− 1.35 (− 1.67; − 1.03)	− 0.35 (− 0.60; − 0.09)	0.0000***
LISAT-9	0.08 (− 0.01; 0.17)	0.15 (0.07; 0.22)	0.00 (− 0.05; 0.05)	0.0026**
HAQ-20	− 0.24 (− 0.32; − 0.15)	− 0.16 (− 0.21; − 0.11)	0.00 (− 0.07; 0.07)	0.0001***

Post-hoc comparison results (*p* value)				
	1 versus 2	1 versus 3	2 versus 3	
Laitinen questionnaire	0.2965	0.0000***	0.0000***	
Pain VAS	1.0000	0.0000***	0.0002***	
LISAT-9	0.2134	0.2748	0.0024**	
HAQ-20	1.0000	0.0003***	0.0038**	

*p*—Probability value calculated using Kruskal–Wallis test.

Assessment of the long-term effects showed clearly visible positive changes in all the measures only in the spa group. Results of post-hoc comparison tests showed that long-term effects identified in spa group differed significantly from the effects observed in outpatient treatment and control groups. Outpatient treatment group differed significantly from the controls only with regard to the scores in Laitinen Questionnaire (Table 5).

**Table 5:** Long-term effects in the groups studied.

Long-term therapeutic effect	Spa group (1)	Outpatient treatment group (2)	Control group (3)	*p*
	Mean therapeutic effect (95% c.i.)			
Laitinen questionnaire	− 3.41 (− 3.99; − 2.83)	− 0.88 (− 1.43; − 0.32)	0.08 (− 0.25; 0.41)	0.0000***
Pain VAS	− 2.77 (− 3.26; − 2.28)	− 0.28 (− 0.59; 0.04)	− 0.17 (− 0.42; 0.08)	0.0000***
LISAT-9	0.31 (0.19; 0.43)	0.02 (− 0.06; 0.10)	− 0.02 (− 0.08; 0.04)	0.0000***
HAQ-20	− 0.39 (− 0.48; − 0.29)	− 0.03 (− 0.11; 0.05)	0.02 (− 0.08; 0.12)	0.0000***

Post-hoc comparison results (*p* value)				
	1 versus 2	1 versus 3	2 versus 3	
Laitinen questionnaire	0.0000***	0.0000***	0.0334*	
Pain VAS	0.0000***	0.0000***	1.0000	
LISAT-9	0.0005***	0.0000***	1.0000	
HAQ-20	0.0000***	0.0000***	1.0000	

*p*—Probability value calculated using Kruskal–Wallis test.

**Figure 2:**
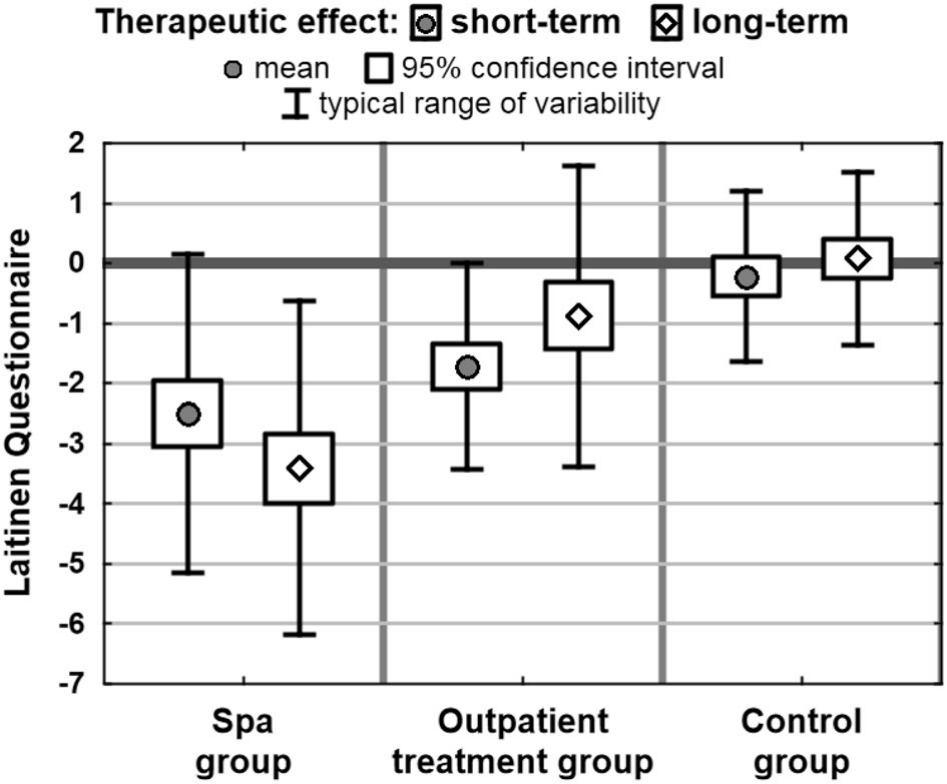
Mean changes in the scores on Laitinen Questionnaire in the groups.

**Figure 3:**
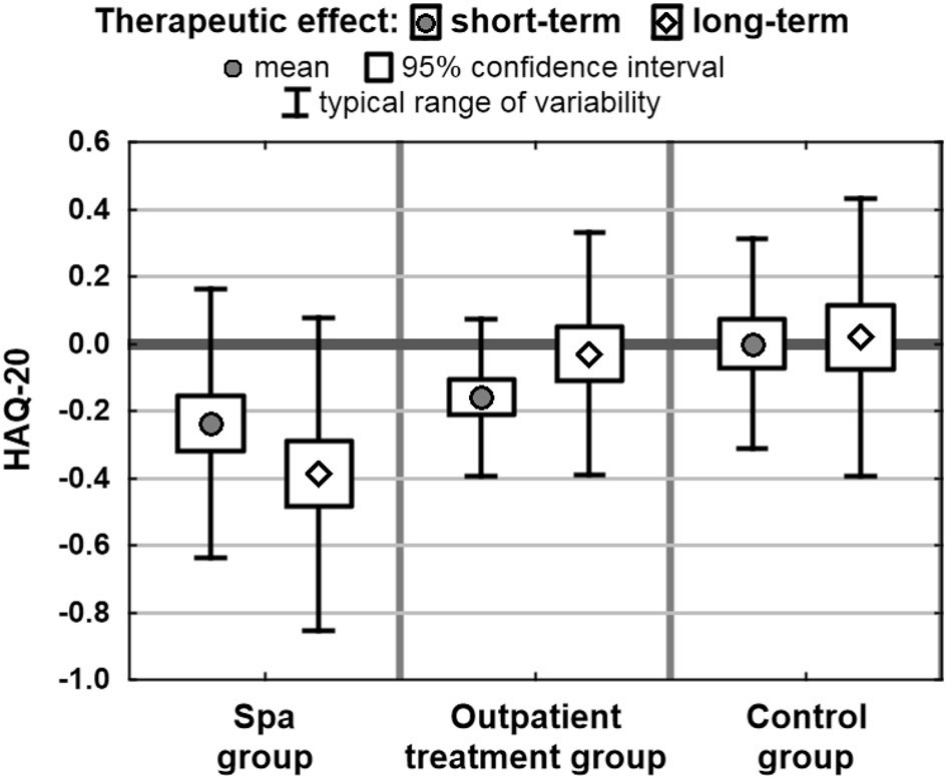
Mean changes in the scores in HAQ-20 in the groups.

Far better short- and long-term effects, reflected by the changes in the scores on Laitinen scale and in HAQ-20 were observed in the spa group. The related changes in the control group were insignificant (Figs. 2 and 3). Here it should be pointed out that the assessment of differences between the cohorts was verified for the effect of other factors using regression analysis. However, the type of therapy was still the key factor in the models which also took into account the potential impact of age, BMI, location of pain, disease duration and place of residence on the effects of therapy. Additionally, assessment of the effects of therapy in individuals receiving treatment in health resorts produced similar results to those acquired using univariate analysis, therefore the study presents only the elementary results (Tables 4 and 5).

The findings show that in the spa group the long-term changes in all the measures were statistically highly significant. The assessment in the outpatient treatment group showed highly significant long-term changes only in the scores achieved in Laitinen Questionnaire. In contrast, no significant long-term changes in the relevant measures were found in the control group (Table 6).

**Table 6:** Short- and long-term effects in the groups studied.

Measure	Mean effect of therapy (with Wilcoxon test result)					
	Spa group		Outpatient treatment group		Control group	
	Short-term	Long-term	Short-term	Long-term	Short-term	Long-term
Laitinen questionnaire	− 2.49	− 3.41	− 1.71	− 0.88	− 0.21	0.08
	(0.0000***)	(0.0000***)	(0.0000***)	(0.0066**)	(0.3463)	(0.7377)
VAS	− 1.66	− 2.77	− 1.35	− 0.28	− 0.35	− 0.17
	(0.0000***)	(0.0000***)	(0.0000***)	(0.1392)	(0.0143*)	(0.1429)
LISAT-9	0.08	0.31	0.15	0.02	0.00	− 0.02
	(0.0145*)	(0.0000***)	(0.0001***)	(0.6907)	(0.3638)	(0.3722)
HAQ-20	− 0.24	− 0.39	− 0.16	− 0.03	0.00	0.02
	(0.0000***)	(0.0000***)	(0.0000***)	(0.6535)	(0.9230)	(0.7312)

*p*—Probability value calculated using Wilcoxon test.

## Discussion

The present study showed that complex spa therapy programs applied to individuals with osteoarthritis of the spine, in the patients’ opinion, produce desirable effects which are maintained for a minimum of six months.

Spa therapy, an important element of complex non-pharmacological treatment, is commonly applied in Europe and worldwide in a variety of rheumatic diseases, e.g., in degenerative disorders affecting the joints and the spine^12,21–23^. Spa therapy leads to reduced pain, improved efficiency in daily life, and increased resistance to stress^11,12,17,23^. According to WHO early old age starts at 60 years^24^. Those aged 65+ constitute approximately 8.3% of the global population, and in this group degenerative diseases of the peripheral joints and the spine are observed at a rate exceeding 60%^3^. The subjects enrolled for the present study on average were aged 67.3 years. Comparative analyses focused on effectiveness of spa therapy provided in health resorts in relation to outpatient treatment with no balneotherapy, and changes taking place in a corresponding period of time in a control group receiving no intervention.

In our study assessment of short-term effects showed reduced pain, improved functional efficiency and greater life satisfaction in both the spa group and the outpatient treatment group. The observed changes were comparable in these two groups. Similar conclusions related to short-term effects were reached by authors of the related literature review^25,26^.

In our opinion, the similarities in the short-term effects observed in the spa therapy group and outpatient treatment group may be explained by the fact that adequately designed complex physiotherapy as well as physiotherapy supplemented with balneotherapy procedures generally produce immediate positive effects.

The current findings related to long-term effects reflected by reduced pain as well as improved functional efficiency and life satisfaction suggest that spa treatment produces better and more sustainable therapeutic outcomes in elderly patients with osteoarthritis of the spine, compared to outpatient physiotherapy. The current findings are even more significant given the fact that the highest rate of obesity was identified in the spa therapy group, and this factor tends to adversely impact effectiveness of rehabilitation and treatments designed to reduce pain. This most probably may be linked to overall effectiveness of complex spa therapy administered on the spot in health resorts. Outpatient physiotherapy alone does not involve change in one’s lifestyle and in one’s diet, and it does not provide an opportunity to take a break from family duties and responsibilities at work, or to benefit from active terrain therapy, climate therapy and health education. The control group was not receiving any systematic treatments, and as it was anticipated, these subjects achieved the best results in the first assessment. No significant short- or long-term changes were identified in this group. Although, the second survey showed improvement in the Pain VAS measurement, this effect was not confirmed by the scores on Laitinen scale. The differences in self-reported assessment of pain may result from the fact that the eleven-item VAS (0–10) is a more sensitive tool than the five-item Laitinen scale (0–4). Numerous studies have demonstrated effectiveness of spa therapy reflected by reduced pain, improved functional efficiency and better quality of life. Good effects were identified in studies which did not involve control groups^1,3,4,27^. In other studies, better effects were found in spa therapy groups compared to outpatient treatment groups^2,9^ and compared to non-intervention control groups^6,10^. Angioni et al. showed positive effects of inpatient spa therapy on pain severity and functional status, but the authors did not observe improvement in the quality of life^16^. Other researchers reported greater improvement as a result of inpatient spa therapy compared to outpatient spa therapy and non-intervention, although effectiveness of the outpatient spa therapy was also satisfying^12^. Likewise, Yücesoy et al. reported significantly better results in a group receiving inpatient spa therapy compared to outpatient spa therapy, but they also emphasised that outpatient spa therapy produces satisfying effects and may be an alternative to inpatient spa therapy^28^. Positive effects of balneotherapeutic factors on treatment outcomes were also reported in other studies where greater improvements were observed in outpatient treatment groups receiving balneotherapy compared to outpatient groups receiving treatments without balneotherapy^5,8,19,20^. A review of the related literature also shows that spa therapy is effective in reducing pain, improving functional efficiency and quality of life in patients with rheumatic disorders, including osteoarthritis of the spine^29,30^.

Effects of spa therapy are maintained for some time after the end of the treatment^17^. Healthy habits acquired during the stay in a health resort contribute to that^10^. According to studies by other authors, effects of spa therapy were maintained at the follow-up after 4 weeks^12^, after 9 weeks^5^, after 10 weeks^8^, after 3 months^2,20^, after 15 weeks^18^, after 4 months^6^ and after 6 months^31^. In the current study better scores reflecting positive effects of spa therapy were observed after 6 months, compared to the non-intervention control group and the group receiving outpatient treatment; in the latter long-term improvement was only reflected by the scores in Laitinen Questionnaire.

We decided to carry out the study in four different health resorts in south-eastern Poland because the composition and properties of the natural resources applied in balneotherapy are similar in that whole area. Furthermore, Polish health resorts are required to comply with certain standards in treatment of disorders accompanied with spinal pain, balneotherapy being one of the components of such treatments. At the initial stage of the study, in order to control for the confounding factors it was determined that the type of therapy administered in the specific health resorts did not significantly differentiate the effects of treatment.

During the initial period of spa therapy problems related to pain may temporarily become aggravated^17^. As a rule, however, the therapy is well tolerated, as a result of which it can be applied multiple times^17,18,21^. Other researchers reported such undesirable events as: more severe pain, increased blood pressure, weakness, allergic reaction and higher temperature. These symptoms usually disappeared after a few hours or a few days^1,30,31^. A literature review showed that only 1% of patients receiving balneotherapy had to discontinue the treatment due to adverse effects^30^. In the current study more severe pain was reported by two subjects and excessive decrease in blood pressure by one subject in the therapy group.

The authors of a literature reviews emphasise drawbacks of the related research, i.e., small study groups, inhomogeneity of the therapies investigated as well as insufficient duration of observation following the therapy^30,32^. Furthermore, because of the complex nature of spa therapies and the characteristic features of the natural materials, it is often impossible to apply a placebo therapy in the control group^6,12,33^. Another limitation reported in the related research lies in the fact that a significant percentage of subjects refuse to participate in the follow-up assessments. For example, in a study by Puszczałowska- Lizis the survey response rate at the follow-up after three months amounted to 61% in the study group and to 50% in the control group^2^. In the present study the entire research procedure was completed by 89% of the subjects in the spa group, 80% of those in the outpatient treatment group and 75% of the controls. Limitations of the current study are related to the specificity of spa therapy in health resorts. Blinding was not applied in the specific groups because of the nature of the therapy and the research protocol adopted. Randomisation, blinding of the participants and placebo type interventions usually are not feasible in research focusing on balneotherapies. Furthermore, it would be unethical to deprive patients of the treatment in order to enable comparative analyses. In view of the above the current study was an attempt to assess combined effects of the complex factors existing and applied in health resorts. The study applied methods of survey and subjective self-assessment, as a result of which it was possible to conduct the second assessment one month after the end of the therapy, and the third assessment six months after the end of the therapy, by means of phone calls.

Environmental factors and climate contribute to the effectiveness of spa therapy^3^. The current study was carried out in health resorts located in south-eastern Poland, where patients can benefit from heliotherapy and climate therapy throughout the year because of the excellent solar and wind related characteristics^34^. A study by Lewicka et al. showed improved health status in 82% of patients after they received treatment in health resorts located in south-eastern Poland^35^.

Spa towns in south-eastern Poland have for many years been attracting patients from all over Poland and from abroad, owing to their natural medicinal and scenic assets. The current findings suggest that rehabilitation and recreation in the climate of south-eastern Poland can be recommended as an effective and affordable therapy to individuals with osteoarthritis of the spine. In order to enable promotion and financing of this form of therapy it is necessary to present supporting evidence confirming the effectiveness of spa therapy provided in health resorts, in accordance with EBM requirements.

## Conclusions

In conclusion, spa therapy may reduce pain, it appears to improve functional efficiency and to increase the level of life satisfaction in patients with osteoarthritis of the spine. These effects are likely to be sustained for six months. As regards long-term benefits, spa therapy seems to be more effective long-term, compared to outpatient treatment.

## Material and methods

### Participants

A prospective study, carried out in the period from April 2019 to June 2020, involved three separate groups. All the participants ranged in age between 60 and 75 years and had been diagnosed with osteoarthritis of the spine, based on both medical and imaging examinations. During the first assessment, all the participants reported they experienced pain, with VAS ratings other than 0 score. Individuals taking anti-inflammatory steroid drugs or those who had received spa therapy or physiotherapy up to 6 months before the start of the study were excluded. Before the first assessment all the participants gave their written consent to take part in the study, and were informed that at each stage they could withdraw from the study without stating any reasons. Since it was impossible for the authors of the study to determine the type of treatment received by the participants, the randomisation procedure was carried out within the specific groups of patients: spa group, outpatient treatment group and control group. After the subject was recruited for a specific group, randomisation was performed using the ‘coin-toss method”. Heads meant inclusion into and tails meant exclusion from the study.

Ultimately 302 subjects took part in the first survey:

- Spa group-102 patients receiving treatment in health resorts in south-eastern Poland (Horyniec Zdrój, Polańczyk Zdrój, Iwonicz Zdrój, Rymanów Zdrój); (Table 7).
- Outpatient treatment group—100 patients receiving treatment at ambulatory physiotherapy clinics in south-eastern Poland.
- Control group- 100 individuals who did not participate in any rehabilitation programs during the observation period.

**Table 7:** Characteristics of the health resorts in south-eastern Poland^34,36,37^.

Health resort	Climate	Bioclimate profile
Horyniec Zdrój, 260 m a.s.l	Typical for lowlands	Mildly stimulating bioclimate; good conditions related to temperatures, precipitation and winds; the warmest region of Poland
Polańczyk Zdrój, 440 m a.s.l	Submontane and montane	Varied bioclimate conditions; submontane health resort next to a massive lake
Iwonicz Zdrój, 410 m a.s.l	Typical for submontane areas with features of continental climate	Moderate bioclimate, strongly stimulating at times
Rymanów Zdrój, 335 m a.s.l	Submontane and montane with predominant features of continental climate	Moderately and at times strongly stimulating bioclimate; submontane health resort in a valley; very good solar conditions

In the spa group and in the outpatient treatment group the survey was conducted during the first three days of the therapy program, one month after the end of the program, and six months after the end of the therapy program. In the control group, the survey was carried out at the same time intervals. Assessment 2 and 3 were conducted over the phone.

### Intervention

All the subjects in the spa group participated, on a daily basis, in morning exercise sessions (15 min) and in group exercise sessions designed for patients with spine disorders (30 min). Individual exercise, based on a neurophysiological approach, was applied in the case of patients with severe pain (30 min). Active terrain therapy (~ 60 min) was recommended to those with milder conditions. The physical therapies applied included conventional TENS (30 min, 100 Hz, 100 µs), as well as magnetotherapy (30 min, 3 mT, 15 Hz) or low-level laser therapy (LLLT, 400 mW, 8 J/point) and classic massage (20 min). The balneotherapies applied included: peloid therapy (20 min, local compresses, 42 °C), individual bath in mineral water (20 min, 36–38 °C) and crenotherapy. The mineral waters used in the treatments included: “hydrogen-sulphide and inorganic sulphide water”, and “chloride-hydrogen-carbonate-sodium, iodide, and acidulous water”. Specification of “hydrogen-sulphide and inorganic sulphide water” is Na^+^, K^+^, Li^+^, Ca^2+^, Mg^2+^, Fe^2+^, F^−^, Cl^−^. Mineralization of this water is 710–820 mg/dm^3^. Hydrogen sulphide level is 34.7–49.6 mg/dm^3^. Specification of “chloride-hydrogen-carbonate-sodium, iodide, and acidulous water” is Na^+^, K^+^, Li^+^, Ca^2+^, Mg^2+^, Fe^2+^, Sr^2+^, Ba^2+^, F^−^, Cl^−^, Br^−^, J^−^. Mineralization of this water is 10,806.3974 mg/dm^3^. The specification of the peloids used is as follows: (a) solid content (non-degraded) ca. 1%, (b) Von Post Humification Scale: H6 or H7, (c) pH: 6.06–6.26, (d) water content: 89.6–90.3%, (e) organic substances: 96.48–95.07% of dry weight, (f) inorganic substances: 3.52–4.90% of dry weight, and (g) silica: 0.03–0.05% of dry weight.

All the patients in ambulatory care received treatment programs provided in their place of residence; the program comprised individual exercise based on neurophysiological approaches (30 min), classic massage (20 min), conventional TENS (30 min, 100 Hz, 100 µsec), magnetotherapy (30 min, 3 mT, 15 Hz) or LLLT (400 mW, 8 J/point).

Both the spa therapy and the outpatient treatment were continued for three weeks, five days a week (from Monday to Friday) with a total of 15 sessions. The controls during the observation period were only allowed to use analgesic pharmacotherapy in emergency.

### Outcome measures

Intensity of pain was assessed using Visual Analogue Scale, where 0 rating corresponds to no pain, and 10 points reflect the most severe pain. Modified Laitinen Pain Questionnaire comprises questions related to intensity of pain, frequency of pain, frequency of using painkillers and level of physical activity. The rating for each of these factors is between 0 and 4 points. The final score is in the range from 0 to 16 points. A higher score reflects more severe pain and greater difficulties in daily life^38^. Assessment was also carried out using Life Satisfaction Questionnaire (LISAT-9). The participants use a scale from 1 to 6 points to rate the quality of their life as a whole, their self-care abilities, leisure situation, vocational and financial situation, sexual life, partner relationship, family life, and contacts with friends. The highest score reflects the best subjective assessment in a given domain^39^. Participants’ self-reported health status and level of disability were measured using Health Assessment Questionnaire (HAQ-20). The tool comprises eight sections related to: dressing, changing one’s position, eating, walking, activities related to hygiene, reaching and griping and general daily activities. In total the questionnaire comprises 20 questions, with responses corresponding to scores ranging from 0 to 3, where 0 rating reflects no difficulties in performing a given activity, while a score of 3 reflects inability to perform a given activity. Arithmetic mean for the scores in all the sections constituted the overall score which was in the range from 0 (no limitations) to 3 (maximum limitations)^40^.

### Statistical methods

The differences in the distributions of Laitinen, VAS, LISAT-9 and HAQ-20 measures in the assessments before and after the rehabilitation program, as well as the differences in effects of the rehabilitation program between the three groups subject to comparative analyses were evaluated for statistical significance using Kruskal–Wallis test. The nonparametric test was applied due to the lack of the normal distribution of the measures in question. However, as verified, the use of parametric tests would have produced very similar *p* values. In this situation it was reasonable to present the average level of a given measure in the assessment before and after the therapy, as well as effects of the therapy in the form of the mean value (with 95% confidence interval). Additionally non-parametric post-hoc comparisons were made, if the results of Kruskal–Wallis test were significant. Assessment of the differences between the groups was verified for impact of other factors (age, BMI, duration of disease, location of pain, place of residence) using regression analysis. This way it was possible to exclude apparent differences between the groups, resulting from their different characteristics. Results of regression analysis are not reported in the article because they simply confirm results of the univariate analysis. Significance of the effects of the therapy in each group was examined using Wilcoxon test. In line with the commonly adopted approach, the differences for which *p* value was below 0.05 were considered statistically significant. Such results are marked with * symbol. Additionally, the marking **, and *** was applied in the case of the results where *p* was below 0.01 or 0.001. The calculations were carried out using STATISTICA v. 13 software.

### Ethical approval

The study was conducted in accordance with the Declaration of Helsinki, and the protocol was approved by the Ethics Committee of University of Rzeszow (Resolution No. Nr 7/04/2019 dated 11/04/2019). Participants gave written informed consent before data collection began.

## Data Availability

The datasets generated during and/or analysed in the course of the current study are available from the corresponding author on reasonable request.
